# Editorial: Wood decomposition: Mechanisms and prevention strategies

**DOI:** 10.3389/fpls.2022.1081131

**Published:** 2022-11-23

**Authors:** Emil E. Thybring, Lisbeth G. Thygesen, Gry Alfredsen, Maria Fredriksson

**Affiliations:** ^1^ Bioresource Chemistry and Technology, Section for Forest, Nature and Biomass, Department of Geosciences and Natural Resource Management, University of Copenhagen, Frederiksberg, Denmark; ^2^ Division of Forest and Forest Resources, Department of Wood Technology, Norwegian Institute of Bioeconomy Research (NIBIO), Ås, Norway; ^3^ Division of Building Materials, Department of Building and Environmental Technology, Lund University, Lund, Sweden

**Keywords:** wood, decomposition, fungal decay, moisture, modeling, durability, construction

## Introduction

The photosynthetic growth of living trees sequesters carbon and predominantly stores it in woody tissue. This storage continues when wood materials are utilized in our society until the materials are eventually decomposed or incinerated. As a biological material, wood can be decomposed by a variety of organisms. The decomposition of wood requires, however, the right environmental conditions of which sufficient moisture is fundamental. Thus, keeping wood dry is a superior strategy for obtaining a long service life, but can be a challenging strategy in outdoor applications. In these situations, the durability of the wood material is important.

This Research Topic focuses on the decomposition of wood in various environments as well as methods for predicting, preventing, or delaying decomposition. The Research Topic includes a study on the role of extractives in brown rot degradation of *P. sylvestris* heartwood (Belt, Harju, et al.). Interestingly, the study indicates that *Rhodonia placenta* is capable of degrading stilbenes in pine heartwood, while *Coniophora puteana* does not have this ability. This difference was reflected in the mass loss observed from the heartwood after five months of exposure to these two brown rot species. This result illustrates that brown rot fungi cannot be assumed to show close to identical degradative pathways or capabilities.

In the study by Tran-Ly, Heeb, et al., the combination of plant oils (linseed and tee tree) and fungal melanin for wood protection is investigated. The focal point of the study is musical instruments, in particular, the ancient wooden wind instrument “The Serpentino”. In such a musical instrument, the condensation of water from the musician’s breath creates a humid microclimate, ideal for the growth of oral bacteria and decay fungi. The study shows a significant anti-bacterial efficacy of the various treatments as well as an effect on the water uptake and dimensional stability of the treated wood.

The study by Belt, Awais, et al. investigates the use of near-infrared imaging for monitoring *P. sylvestris* wood during brown rot decay (*Rhodonia placenta* or *Coniophora puteana*). The data are analyzed and clustered using ANOVA simultaneous component analysis (ASCA) and principal component analysis (PCA). The results confirm that brown rot decay reduces the number of carbohydrates in the wood, while altered lignin remains, and that primarily the earlywood is affected by the degradation.

Two papers in this Research Topic provide new knowledge about Eutypa dieback and Esca complex, fungal diseases of grapes that cause large economic losses in vineyards. The study by Schilling, Maia-Grondard, et al. focuses on the white rot fungus *Fomitiporia mediterranea* (a typical symptom of Esca) using physiologic, metabolomic, and proteomic approaches. *F. mediterranea* causes higher mass loss for grapevine than for spruce and beech. Simultaneous decay patterns are demonstrated, and proteomic analyses identify a relative overproduction of oxidoreductases involved in lignin and extractive degradation on grapevine cultures, and only a few differences in carbohydrate-active enzymes. The results partially explain the adaptation of *F. mediterranea* to the structural composition of grapevine wood and suggest that other biotic and abiotic factors should be considered to fully understand the potential adaptation of *F. mediterranea* to its ecological niche.

In the second paper about Eutypa dieback and Esca complex, Perez-Gonzalez, Sebestyen, et al. study oxygen radical-generating metabolites secreted by Eutypa and Esca fungal consortia. Unique metabolites are isolated from the consortia fungi. Some metabolites preferentially reduce iron whereas others are involved in redox cycling to generate hydrogen peroxide. Interestingly, metabolite suites with different functions are produced when fungi are grown separately vs. when grown in consortia. Chelator-mediated Fenton chemistry promoted by metabolites from these fungi allows for the generation of highly reactive hydroxyl radicals. The authors hypothesize that this mechanism may be involved in pathogenicity in grapevine tissue as a causal mechanism associated with trunk wood deterioration/necrosis in these two diseases of grape.

## Author contributions

All authors listed have made a substantial, direct, and intellectual contribution to the work and approved it for publication.

## Conflict of interest

The authors declare that the research was conducted in the absence of any commercial or financial relationships that could be construed as a potential conflict of interest.

## Publisher’s note

All claims expressed in this article are solely those of the authors and do not necessarily represent those of their affiliated organizations, or those of the publisher, the editors and the reviewers. Any product that may be evaluated in this article, or claim that may be made by its manufacturer, is not guaranteed or endorsed by the publisher.

